# MEWAR: Development of a Cross-Platform Mobile Application and Web Dashboard System for Real-Time Mosquito Surveillance in Northeast Brazil

**DOI:** 10.3389/fpubh.2021.754072

**Published:** 2021-10-27

**Authors:** Aisha Aldosery, Anwar Musah, Georgiana Birjovanu, Giselle Moreno, Andrei Boscor, Livia Dutra, George Santos, Vania Nunes, Rossandra Oliveira, Tercio Ambrizzi, Tiago Massoni, Wellington Pinheiro dos Santos, Patty Kostkova

**Affiliations:** ^1^Centre for Digital Public Health & Emergencies, Institute for Risk and Disaster Reduction, University College London, London, United Kingdom; ^2^Department of Atmospheric Sciences, Institute of Astronomy, Geophysics and Atmospheric Sciences (IAG), University of São Paulo, São Paulo, Brazil; ^3^Recife City Hall, Recife, Brazil; ^4^Campina Grande City Hall, Campina Grande, Brazil; ^5^Department of Systems & Computing, Federal University of Campina Grande, Campina Grande, Brazil; ^6^Department of Biomedical Engineering, Federal University of Pernambuco, Recife, Brazil

**Keywords:** mobile technology, real-time, surveillance, mosquito, environmental surveillance agents, environmental health agents

## Abstract

Mosquito surveillance is a crucial process for understanding the population dynamics of mosquitoes, as well as implementing interventional programs for controlling and preventing the spread of mosquito-borne diseases. Environmental surveillance agents who performing routine entomological surveys at properties in areas where mosquito-borne diseases are endemic play a critical role in vector surveillance by searching and destroying mosquito hotspots as well as collate information on locations with increased infestation. Currently, the process of recording information on paper-based forms is time-consuming and painstaking due to manual effort. The introduction of mobile surveillance applications will therefore improve the process of data collection, timely reporting, and field worker performance. Digital-based surveillance is critical in reporting real-time data; indeed, the real-time capture of data with phones could be used for predictive analytical models to predict mosquito population dynamics, enabling early warning detection of hotspots and thus alerting fieldworker agents into immediate action. This paper describes the development of a cross-platform digital system for improving mosquito surveillance in Brazil. It comprises of two components: a dashboard for managers and a mobile application for health agents. The former enables managers to assign properties to health workers who then survey them for mosquitoes and to monitor the progress of inspection visits in real-time. The latter, which is primarily designed as a data collection tool, enables the environmental surveillance agents to act on their assigned tasks of recording the details of the properties at inspections by filling out digital forms built into the mobile application, as well as details relating to mosquito infestation. The system presented in this paper was co-developed with significant input with environmental agents in two Brazilian cities where it is currently being piloted.

## Introduction

Mosquito-borne diseases place a huge public health burden on the inhabitants of tropical regions. In Brazil, diseases such as zika, dengue, and chikungunya are arboviruses transmitted primarily by the *Aedes* mosquito which are endemic in Latin America. Other vectors like the *Culex* transmit diseases such as West Nile fever and Japanese encephalitis (1, 2). Mosquito-control surveillance is an essential component of the health interventions initiated by the public health authority to control and prevent the spread of diseases. Environmental surveillance agents play an indispensable role in controlling mosquitoes by identifying and treating infected dwellings. They do this by scheduling routine household visits to make sure homes and backyards have no mosquito hotspots. During the visits, the workers record the data on paper forms designed by the local community (3). Despite the success of the agents in mosquito-control surveillance, the traditional paper-based system and the process of transcribing the data onto a computer system is a time-consuming and error-prone undertaking, which potentially yields low-quality data and causes delays in the analysis. Using mobile technology is therefore considered an effective way to support health agents in collecting and reporting data in real time (4).

There is increasing interest in the use of mobile applications for capturing and transferring data in the field of public health research, including research into mosquito-borne diseases, where mobile applications have been adopted for disease surveillance or vector surveillance (5). The applications vary with respect to their primary functionality and features, and include text-messaging outbreak alert apps (6), data collection apps (7, 8), and vector surveillance apps (5). Most of the data collection applications were developed for public use for the recording of symptoms (4). A small number have been developed for vector surveillance. For example, VectorPoint (9) is a mobile surveillance application for the vector that causes Chagas disease in Arequipa, Perú. The application provides a risk map based on data collected during fieldwork. To the best of our knowledge, there are two existing vector surveillance mobile applications dedicated to mosquito surveillance: Chaak (10) captures data related to the immature stages of dengue virus mosquito vectors in Mérida, México; VazaDengue (11) integrates social media with citizen science to guide surveillance agents in controlling mosquitoes. Chaak, a cellphone-based system, is comparable to the system we propose here. Chaak provides two main components: a platform that enables managers to coordinate and manage health workers, and a mobile application to enable health workers to collect field data on the presence or absence of mosquitoes (10). One limitation of the Chaak system is that new property addresses can only be recorded by managers; health agents are unable to change property details or add extra information. The system also suffers from incompatibility with various mobile operating systems and is unsuitable for large-scale implementation.

In Recife and Campina Grande, two cities in Northeast Brazil, vector control relies on agents from their environmental authorities who known in Recife as Environmental Health and Endemic Control Agents (ASACEs) and Campina Grande as Environmental Health Agents (ASAs) to combat increased mosquito activities. The current process at these institutions involves managers assigning surveillance visit tasks for specific geo-entities (areas and neighborhoods) to the agents, who visit the premises and record data regarding mosquitoes on paper-based forms. The agents go from house to house checking for mosquito breeding habitats in containers holding stagnant water, inside, and around residential premises. Such sites are environmental risk factors for indoor and outdoor mosquito infestation. The agents count the containers holding immature insects and collect information regarding larvae and pupae. This data is then copied onto a computer-based system. The process of transcribing the data is time consuming and prone to errors, resulting in a reduction in accuracy and an increase in vector control response time, and only enabling such information to be analyzed at coarser ecological units as demonstrated in previous research that have used such data (12, 13).

Mobile applications provide high-quality real-time field data and enhance the coordination and performance of surveillance agents. They therefore represent a promising tool in the fight against mosquito-borne diseases. To assist in this fight, and based on guidelines provided by the local communities in Recife and Campina Grande, we developed a cloud-based system (the MEWAR IT system) which improves the surveillance of the mosquito by providing timely and geolocated reports regarding the presence and absence of mosquito (eggs or larva) in properties without colleting any climate data or epidemiological data (individual health information). The name of the research project is called “Mosquito Population Modelling for Early Warning & Public Health Response,” which is abbreviated as MEWAR. Thus, we have chosen to name the tool after it as MEWAR.

The MEWAR system comprises a web-based platform for the management of inspection visits and a cross-platform mobile application for capturing data; to enable dynamic interaction between the platforms, the two components share a database. MEWAR system is considered unique compared to other existing mosquito surveillance systems. It does not depend on social science (citizen science) or social network (i.e., social media such as Twitter, Facebook, etc.) to collect vector-related data (11). It also provides agents with more flexibility in terms of decision-making on the ground, which is crucial for keeping agents engaged with the app. For example, it is not specifying properties to be inspected (10). Rather, it specifies a neighborhood and number of properties for each inspection task; it is not dedicating a path to follow while inspecting properties, which usually other vector surveillance systems do by generating specific routes based on infestation risk or modeling (9). The system also gives control to the agents to add new addresses if not exist rather than limiting this to managers only (10). At the same time, it does not restrict the format of the addresses. Finally, the technical infrastructure and the support of multiple platforms allow large-scale implementation of the MEWAR, as well as make the system adaptable to other different scenarios and settings.

This paper presents the process of designing, building, and developing the MEWAR system and the architecture of the proposed mosquito surveillance system that supports environmental surveillance agents in their routine inspection visits in the two Brazilian cities of Recife and Campina Grande.

The paper has seven main sections. Section System Design and Development Process describes the system design, the co-authoring process, and the process of designing and contextualizing the mosquito surveillance system. Section MEWAR Surveillance System Overview provides the workflow of the system and the interaction with end users. Section Key Features and Functionalities of the Surveillance System describes the key features of the system. Section MEWAR Surveillance System Implementation presents the system's main components and technical details of the system implementation. Section Discussion and Future Work presents a discussion and a reflection on lessons learned by the research team and suggests ways in which the system may be expanded in the future with respect to features and functionality. The final section presents some conclusions.

## System Design and Development Process

The system has been designed and developed over a period of two and half years through an iterative co-creation process in a close collaboration between researchers and software engineers from University College London (UCL) and from two Brazilian universities: the Federal University of Pernambuco (UFPE) and the Federal University of Campina Grande (UFCG). Most importantly, the development has involved a close collaboration and the co-authoring with Brazilian health agents from the environmental surveillance agencies in Recife (ASACEs) and Campina Grande (ASAs). Academics from UFPE and UFCG served as a bridge between the development team in UCL and the environmental surveillance agencies in the two cities. The health agents provided the development team with invaluable information and shared insights into the processes involved in their data collection and fieldwork.

**Figure 1:**
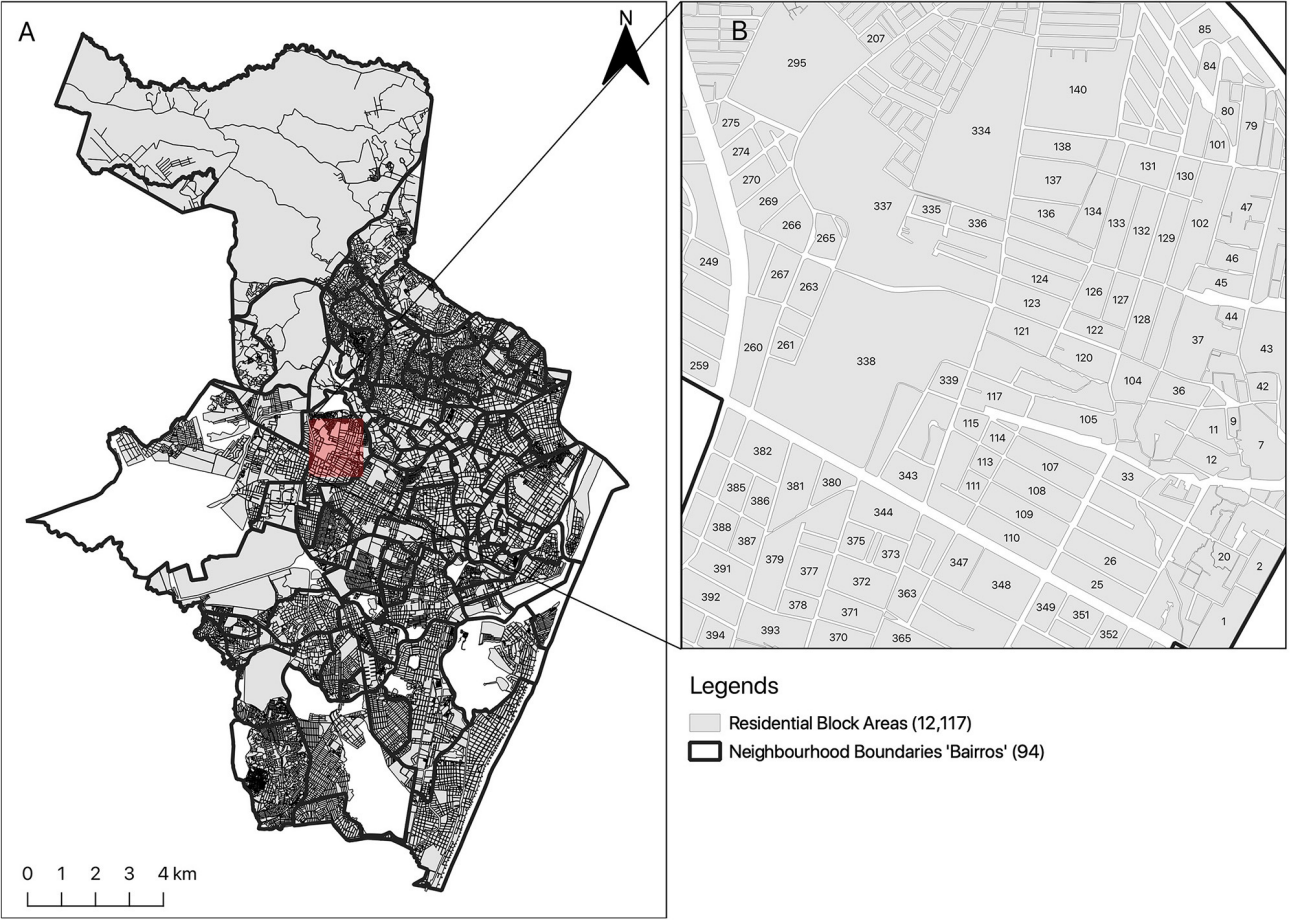
The study area of Recife showing the spatial configuration of the neighborhoods **(A)** and block areas **(B)**.

**Figure 2:**
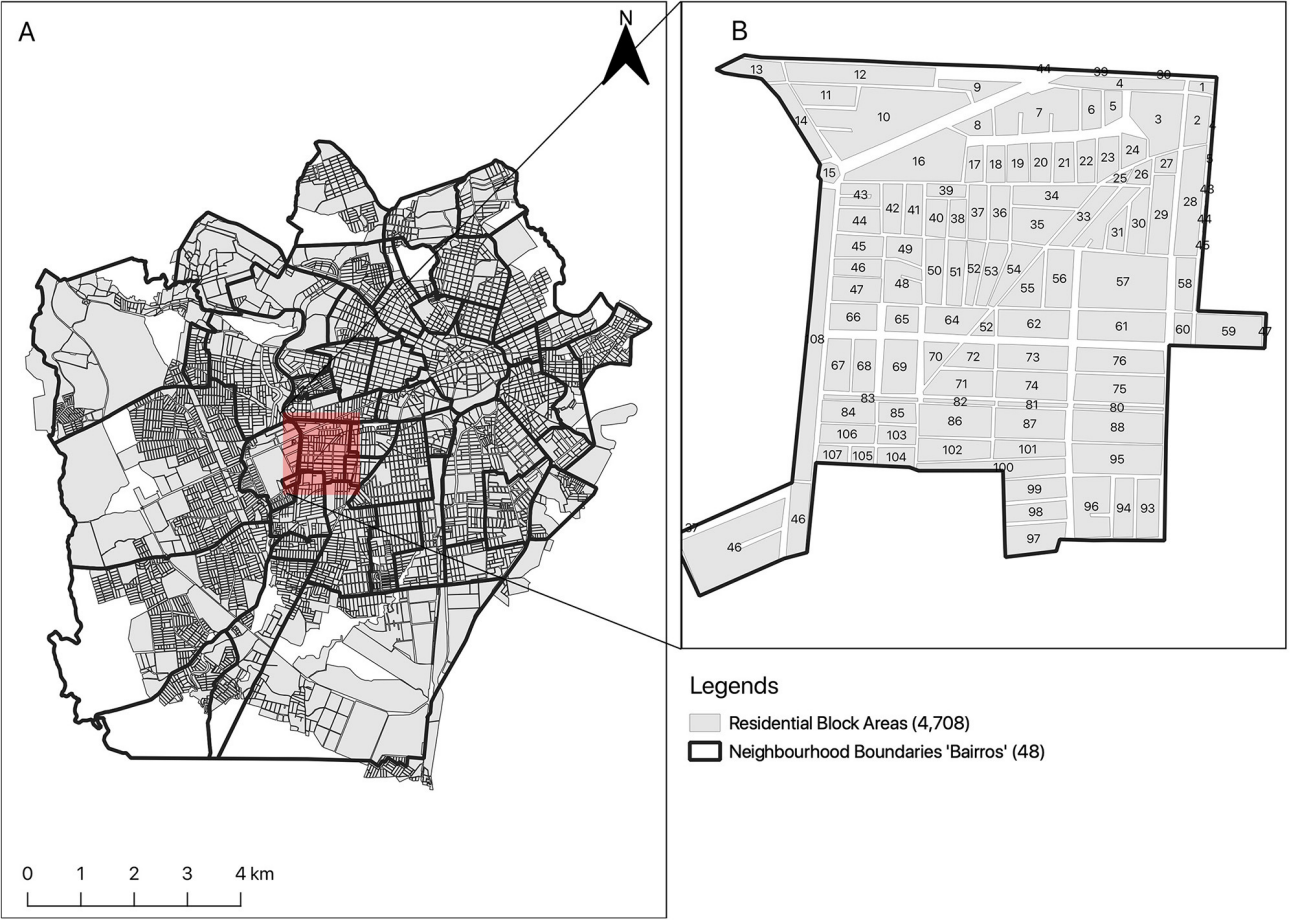
The study area of Campina Grande showing the spatial configuration of the neighborhoods **(A)** and block areas **(B)**.

Figures 1, 2 show the location of Recife (State of Pernambuco), and Campina Grande (State of Paraíba), the two cities in which this study is conducted. The two cities are representative of the mosquito surveillance methods used across all Brazilian cities as defined by the Brazilian Ministry of Health. Recife city is located along the coasts (8°03′S34°52′W), with 1,661,017 inhabitants and 218 k*m*^2^ area, making it one of Brazil's highest population density cities (14). The second site study is Campina Grande (7°13′14.92″S, 35°55′1.32″W) which is also located in Northeast Brazil. It is considered the industrial center of Northeast Brazil and the main technology center in South America. The estimated population in 2021 is 413,830, making it the second-most populous city of Paraíba (15). Since 1995, the state of Pernambuco has been endemic for mosquito-borne disease (dengue virus). In 2015, other mosquito-borne diseases (Zika and Chikungunya viruses) were introduced as well in Pernambuco, which in turn yields, circulate these mosquito-borne diseases within a geographic region (i.e., Northeast Brazil). The co-circulating of these viruses poses a significant challenge to health authorities (16). Climatic conditions (temperature, humidity, rainfall level, etc.,) in Northeast Brazil, including, Recife and Campina Grande, are ideal for mosquito living, making them suitable for mosquitoes abundance, thus, optimal sites for our study objective [i.e., mosquito surveillance; (14, 15, 17)].

### Focus Groups Iterative Development

The development of the app included four focus group discussions with the intended end users (environmental surveillance agencies) from the two cities. The first two discussions were in Brazil, and all focus groups and interview participants provided their informed written consent. The other two were conducted online and all participants gave verbal informed consent. The aim of these sessions was to define user requirements, technical specification, user experience (UX), and user interface (UI) (i.e., look-and-feel), and to assess functionality. The objectives and details of each discussion are described in the following subsections.

#### Focus Group One—In Person in Brazil

The first focus groups were carried out at two study sites, Recife and Olinda in Northeast Brazil, in February 2018. The aim was to brainstorm the vision for mobile mosquito surveillance, involve surveillance agents in the project and start the collaboration and to test the first prototype version of the ZIKA app developed by the team at UCL in 2018 and described in detail by Beltrán et al. (18). During this field trip, one focus group was conducted in each of the two cities. The manager dashboard was presented to the environmental agents, and the mobile app was tested to elicit feedback. Five agents from Recife and three agents from Olinda were given the opportunity to test the mobile app on their phone during the sessions and shared their feedback verbally in the local language, Portuguese, discussions were audio recorded. These responses were later translated into English and a new set of requirements was compiled, which included the following changes: (1) local variations for each city which include customizing some components of surveillance forms to fit each city's needs, as well as managing the system access control by identifying users city; (2) expanding the mosquito forms to include different types of mosquitoes (i.e., Culex); (3) the app should not include the route suggestion which dictates a particular path to follow or mandate which property to surveil feature as the agents prefer to decide themselves and visit properties using their own “blocks” system. The specifications and main functionalities of the manager dashboard were also discussed. Lastly, based on the first focus group, the proposed system was updated and improved for the next iterations of the prototype; the details are described in the following sections.

#### Focus Group Two—In Person in Brazil

The second participatory focus groups were carried out at two study sites in Brazil in August/September 2019. The primary goal was to showcase the initial concept of the new version of the system, including both the manager dashboard and the mobile application for agents. This was an improved iteration of the first version of the gamification app developed in 2018 (18). The aim of the field trip was to engage with the environmental surveillance managers and agents, to assess local conditions and needs, and to understand how the agents carry out their routine inspection visits as dictated by the surveillance forms for capturing mosquito information. The feedback provided during this trip was crucial in helping us to translate the surveillance paper forms into the app and system.

We held three follow-up workshops with the managers and health agents: one in Recife (with managers and agents together) and two in Campina Grande (separate workshops for managers and agents), these workshops were audio recorded and later transcribed into English. The Recife workshop involved 11 participants: 6 environmental managers (1 biologist and 5 veterinary medical practitioners) and 5 health agents. The two Campina Grande workshops were attended, respectively, by 16 environmental managers and 66 health agents. The agents had the opportunity to test a prototype version of the mobile phone application.

During the workshops, we explained the goal of the new version of the system, which primarily functions as a surveillance data collection tool. Mock-ups and other prototypes of the system components (18) were also showcased to the target audiences and end users to prompt discussion regarding the major features and functionalities that could be offered by the tool in order to move toward a digital-based surveillance system. Moreover, two paper forms were distributed across the managers and agents during these workshops to collect their feedback about each page in the dashboard and the app. The managers' form contains 11 questions, six were binary questions (i.e., Yes/No answers), and the rest were about other functionalities they would like to include. On the other hand, the agents' form contains 11 questions; out of that, nine were binary questions (i.e., Yes/No answers), one about their thoughts regarding the app's usability, and lastly, any additional functionalities. Several new features were introduced to enhance the operational flexibility of the proposed tool and to make it adaptable to different local settings and conditions. For example, the agents requested that certain entry fields in the form be customized according to the user's city. The discussions and the collected form answers enabled the research team as well as the developer team to better understand the local context and the needs of end users and to build this learning into the foundations of the system (19).

The environmental surveillance agencies in both cities shared the paper-based mosquito surveillance forms they use, as well as maps of each city which show the spatial configuration of the relevant residential neighborhoods. The development team from UCL translated the forms into English, standardized the two surveillance forms from the two cities, and used participatory GIS in the UK to digitize the paper maps provided by the managers from Campina Grande (see Figure 2); Recife already had digital surveillance maps (see Figure 1). The spatial data will be used to initialize the database with the geographical information and will be integrated into the manager dashboard to enable visualization of areas at high risk of mosquito infestation. The development of this feature is a work in progress.

#### Focus Group Three—Online

As a result of the Covid-19 pandemic, the third focus group discussions were run virtually, online, in September 2020. To make the focus group manageable, each panel was limited to four researchers, two managers, and three health agents. The team from the two cities were met separately via the videoconferencing platform Google Meet for around 3 h; all participants joined using both video and audio. The discussions were conducted in Portuguese and were recorded. Notes were taken simultaneously in English by two reporters. Immediately after the session, these notes were compared and combined into a single English-language summary of the discussion. The main objective of this focus group was to show the first completed version of the system and to receive feedback regarding the system's usability, particularly the system functions and user interface.

**Figure 3:**
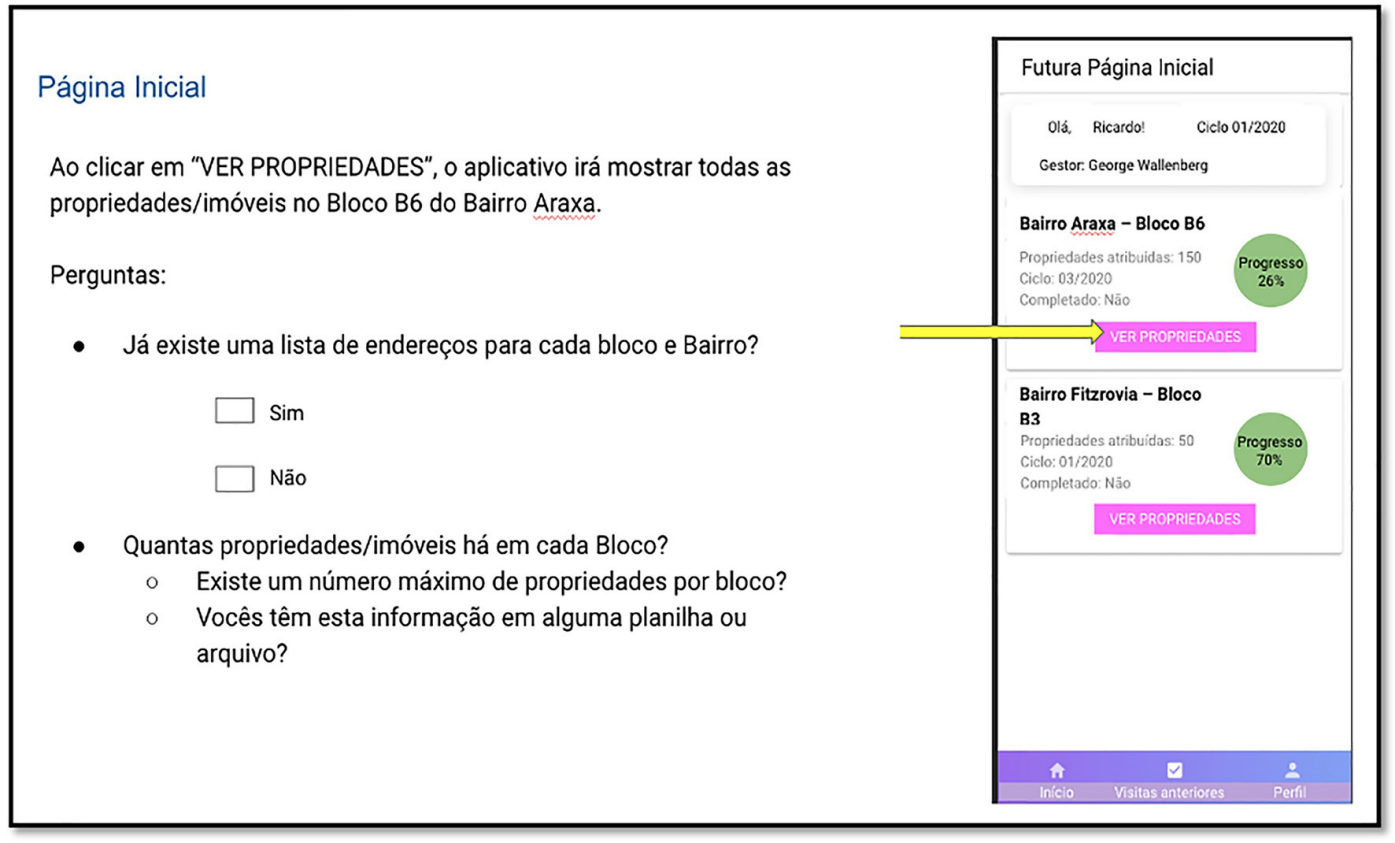
Example of the slide containing a screenshot from the app **(Right)** and the questions (in Portuguese) to be discussed with the participants **(Left)**.

Each focus group began with an explanation of how the session would proceed. Following this introduction, a presentation was delivered to the participants, who were asked to assess whether the system was consistent with their requirements. A series of screenshots and other images of the system prototype were taken to mirror the same order and workflow of the system (Figure 3 shows an example of the focus group presentation). The participants provided feedback regarding interface design, additional features, supported languages, and the structure of the forms, and offered suggestions for possible features on both the manager dashboard and the mobile application for agents.

During this focus group, several new features were introduced to enhance the functionality of the manager's dashboard, such as feeding back and displaying the progress of the agents when conducting field surveillance using the app by showing the number of completed and remaining surveillance visits of each agent. Managers should also be able to add new geographical information onto the database, such as newly formed areas (new residential blocks, micro-regions) and define them according to their corresponding neighborhoods. One suggestion for the mobile app was to have a separate page to view the submitted visits forms (i.e., historical records) by filtering search data according to the name of the area visited, the name of the block, and the date. Other popular suggestions were to add extra fields to some of the pages on the form and to make some fields optional, both of which would provide more flexibility. The design of the mobile application home page was also discussed, and it was agreed that essential components would include a user bio-card and a list of assigned tasks provided by the manager. Finally, suggestions for future development of the system, including increased automation, were documented. Most of the comments regarding the new version were addressed before the final focus group, which was therefore able to focus on testing the system.

#### Focus Group Four—User Acceptance Testing—Online

The final focus groups were run virtually in February 2021. Each of the two discussions was limited to three researchers, one manager, and one agent, and lasted 2 h. It was not possible to have more people from the environmental surveillance agencies as they were occupied with work related to surveillance of Covid-19, which became their priority. Prior to the discussions, the project coordinator arranged the first 3-day testing phase. This included a day of training (conducted online, in Portuguese) for managers and agents during which managers were shown how to access the manager dashboard and agents were shown how to download and install the mobile application on their phone. User guidance and a tour of the system from both perspectives were provided. The manager and agent spent the remaining 2 days testing the system using simulations of in-field scenarios.

After 3 days of testing, the team met for the fourth focus group (conducted in Portuguese and English) using the videoconferencing platform Google Meet lasted 2 h; focus group recorded, all participants joined using both video and audio discuss the user-friendliness of the tool and the overall level of satisfaction with the system. Further feedback was provided on features and functionalities that would increase the system's flexibility. It was stressed that keeping the system flexible and easy to use was vital. One significant suggestion was to integrate the geographical information of the properties with the residential block areas and neighborhoods so that users did not have to enter property details manually. Other suggestions related to some of the jargon used for options in the dropdown list.

Following this final feedback from health workers, the relevant aspects of the system were amended. The results were shared with the participants and the 6-week data collection planned to begin in mid-August 2021 due to the COVID-19 situation in Brazil. The pilot would be conducted in a limited number of neighborhoods in each of the two cities. The principal objective was to evaluate the usability and scalability of the system in a real operation in the field in which agents would visit properties and record the data using both the standard paper forms and the mobile application. The plan is then to pilot the system in a much larger field study as part of long-term research aimed at developing an early warning system that will help to control mosquito infestation.

## MEWAR Surveillance System Overview

**Figure 4:**
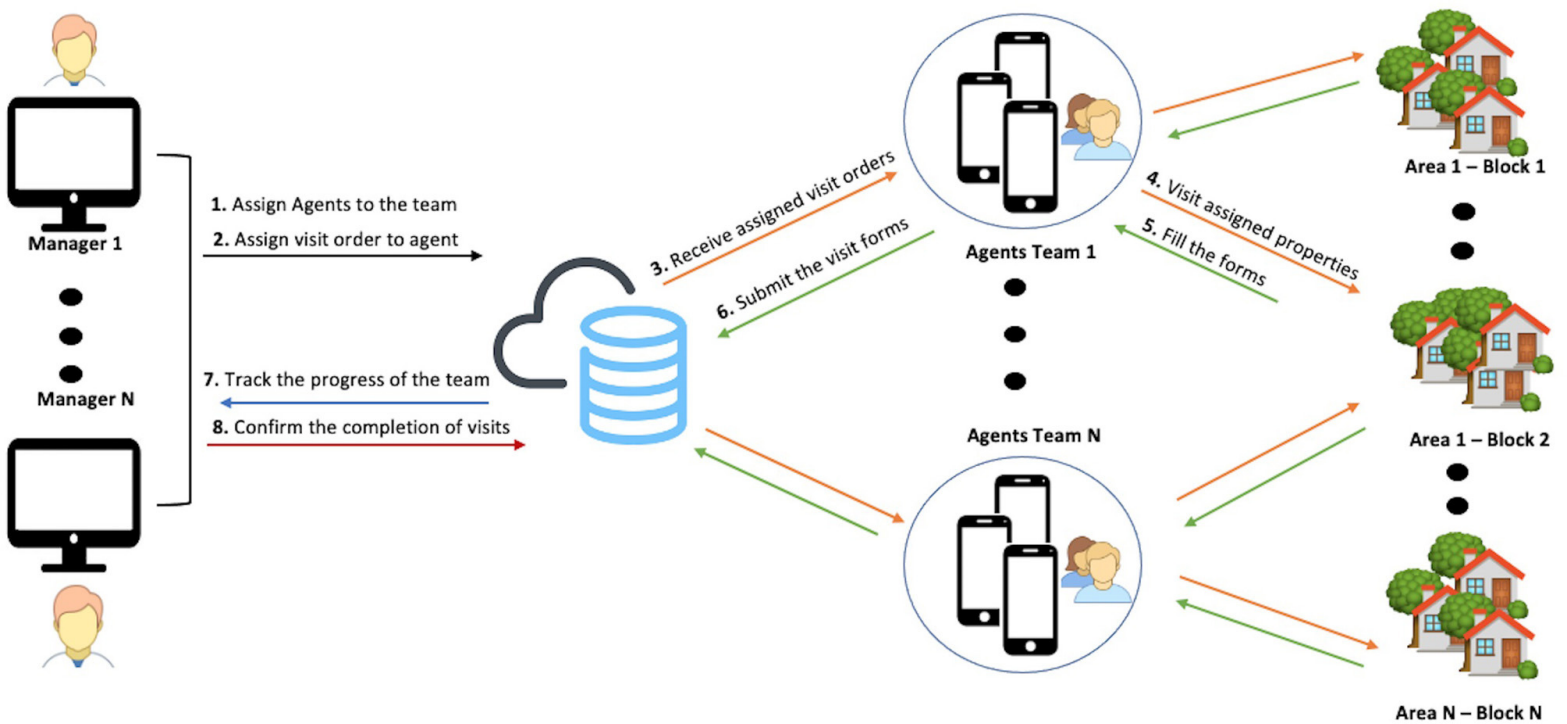
Overview of the proposed MEWAR system.

Surveillance of mosquito is a routine data collection process which requires regular visits to properties to assess any breeding hotspots in water containers and to destroy these and so reduce the infection of humans. The MEWAR surveillance system helps environmental health agencies to conduct this operation digitally. Figure 4 presents an overview of the proposed system and the interaction between the two main components. The system consists of a website, a mobile app, and a cloud server. When allocating agents to routine inspection visits, managers assign an agent to a team and allocate them to a specific area using the manager platform. The system then allows a manager to assign inspection visit tasks to specific geographical entities (area, neighborhoods, and a certain number of properties) to be completed during a particular period. The agent receives the assigned tasks on the mobile application then visits the properties in person and looks for mosquito breeding hotspots in water containers and backyards. The agent records and enters the surveillance data on the digital form using the mobile application. On completion of data entry, the forms are uploaded to a cloud-based database. Managers can track the progress of each agent in real time, and when the assigned visits are completed, can confirm the completion of the task.

## Key Features and Functionalities of the Surveillance System

The proposed MEWAR system offers users two primary services: coordination and supervision of surveillance health agents; and a data collection tool for mosquito surveillance. These services are described in detail below.

### Manager Dashboard: Supervision of Surveillance Agents and Allocation of Tasks

**Figure 5:**
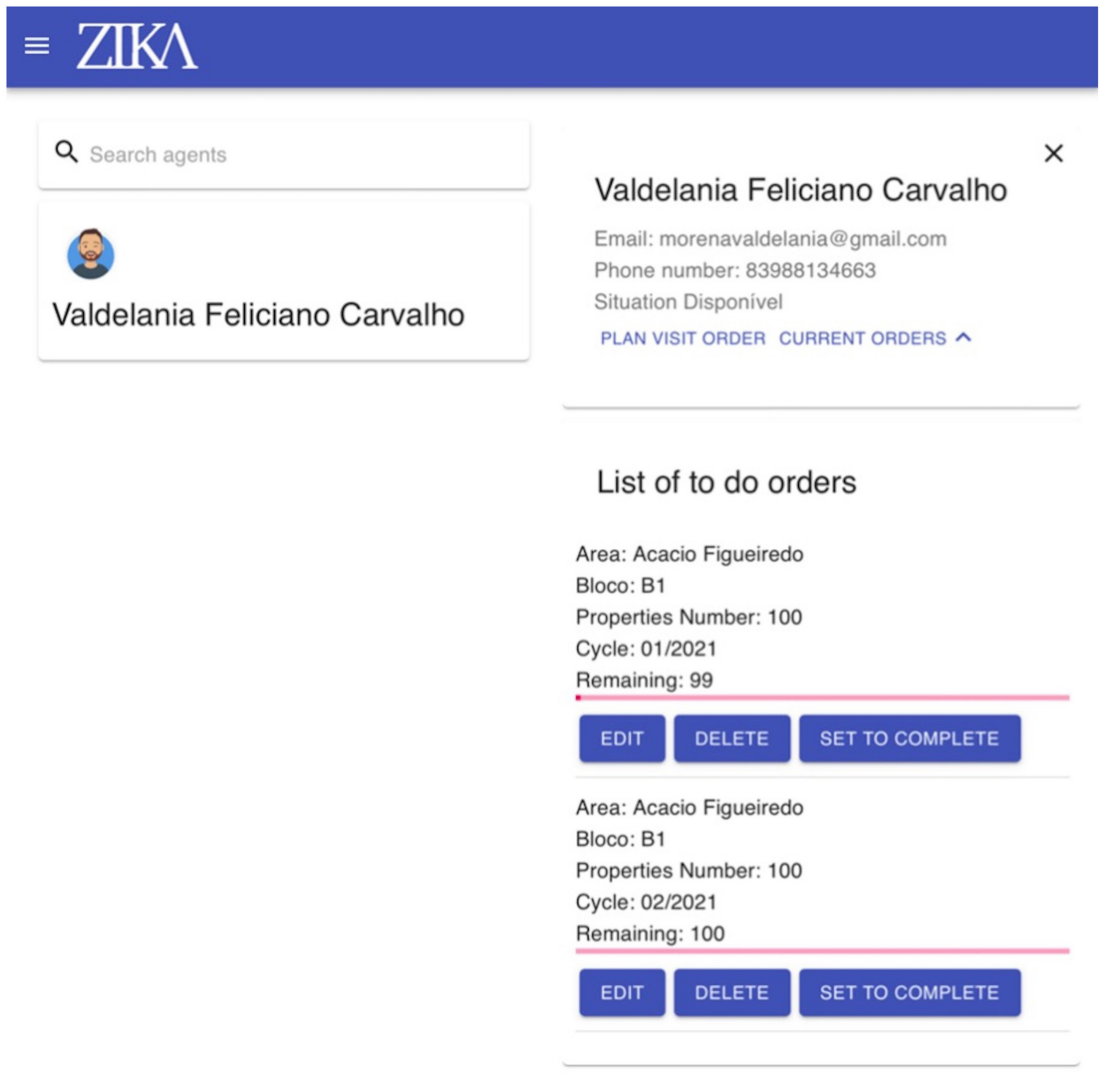
Home page of the managers dashboard showing the interface used by managers for selecting and viewing agent details, and for allocating and assigning tasks to agents (right).

The manager dashboard allows environmental managers to coordinate the routine surveillance visits performed by health agents and to monitor their progress in real time. The dashboard is adaptable to different locations (states, cities) and languages (English, Portuguese). The manager dashboard assigns unique configurations based on their user profile—the user's registered city—in order to adjust and customize the list of areas, neighborhoods, and health agents. Each manager only has access to information relevant to their city and to local agents. Agents are registered to a specific manager and designated to a specific area. Managers create their team by adding agents who work under their supervision. This minimizes the list of agents and accelerates the process of managing and monitoring each agent. Figure 5 shows the manager dashboard home page where the key functionalities (delete, edit, set to complete) and information (list of agents and assigned tasks) are displayed. The domain-specific functionalities that managers have access to are: (1) adding agents to their team; (2) allocating agents to a specific geographical area; (3) creating visit tasks for a specific number of properties in a neighborhood for each agent to be completed within specific time (which precise properties are visited is not dictated by the manager); (4) editing or removing existing tasks (if a task is removed, any form that has been submitted as part of this task will not be deleted); (5) monitoring the progress of the work using the progress bar (which shows the number of forms submitted relative to the total assigned); (6) approving the completion of the assigned tasks; (7) defining new geographical areas and associated neighborhoods. The manager dashboard contains all the information required to assign an inspection visit task, such as geographical data (areas name, neighborhood ID) and a list of all registered agents. Moreover, the dashboard provides real-time progress reports by showing the number of completed visit forms for each assigned task. Hence, it assists the managers in monitoring the progress and assessing the situation on the field in real-time and rapidly making any required decisions (i.e., allocating or reallocating agents for blocks with slower progress and so on). The overarching goal of manager dashboard is to integrate both mosquito surveillance and risk modeling data to understand and predict the areas at high risk of mosquito infestation. Thus, the collected data (through the app) and predictive models will in the long run be able to inform policymakers how to improve surveillance and operations in the field, have evidence for staffing the environmental agencies with agents, and also evidence for investing in better water sanitation in areas where mosquito infestations seem to be a major issue.

### Mobile Application for Health Agents: Data Collection Tool

**Figure 6:**
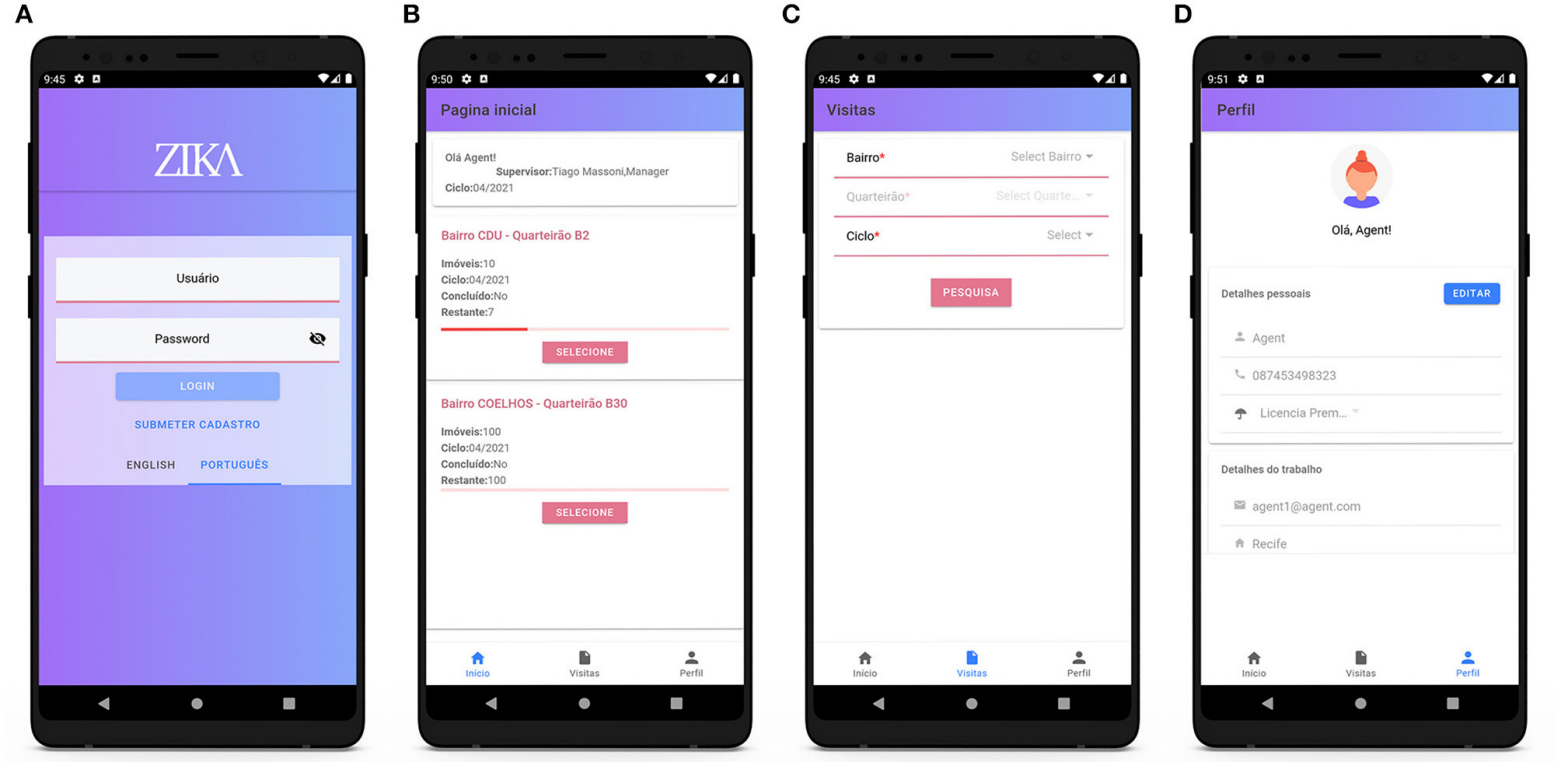
Surveillance agents' mobile application: login page and the three main pages of the application. **(A)** Login page, **(B)** home page, **(C)** past visits, and **(D)** profile page.

The second component of the MEWAR system is a mobile application for the surveillance of mosquito vectors. The main operators of this application are health agents, and its principal functionality regards in-field data entry using built-in digital forms. Another notable feature is the flexibility that allows agents to edit property addresses and add missing ones. Agents who register and log into the app have access to all application functionalities. They receive the tasks assigned to them by their managers and a list of assigned inspection visits is displayed on their home page. The tasks are defined by area name, neighborhood, the number of properties that need to be inspected, and the cycle, which represents the months of the year during which the task is to be carried out (for example, January and February are Cycle 1) (Figure 6). The agent then selects a specific task and starts filling out the surveillance forms. The forms are designed to collect the same data as that collected through the standard paper-based forms currently used by health agents in both Recife and Campina Grande. The forms are classified according to four main categories of data: descriptive, logistical, entomological, and environmental.

**Figure 7:**
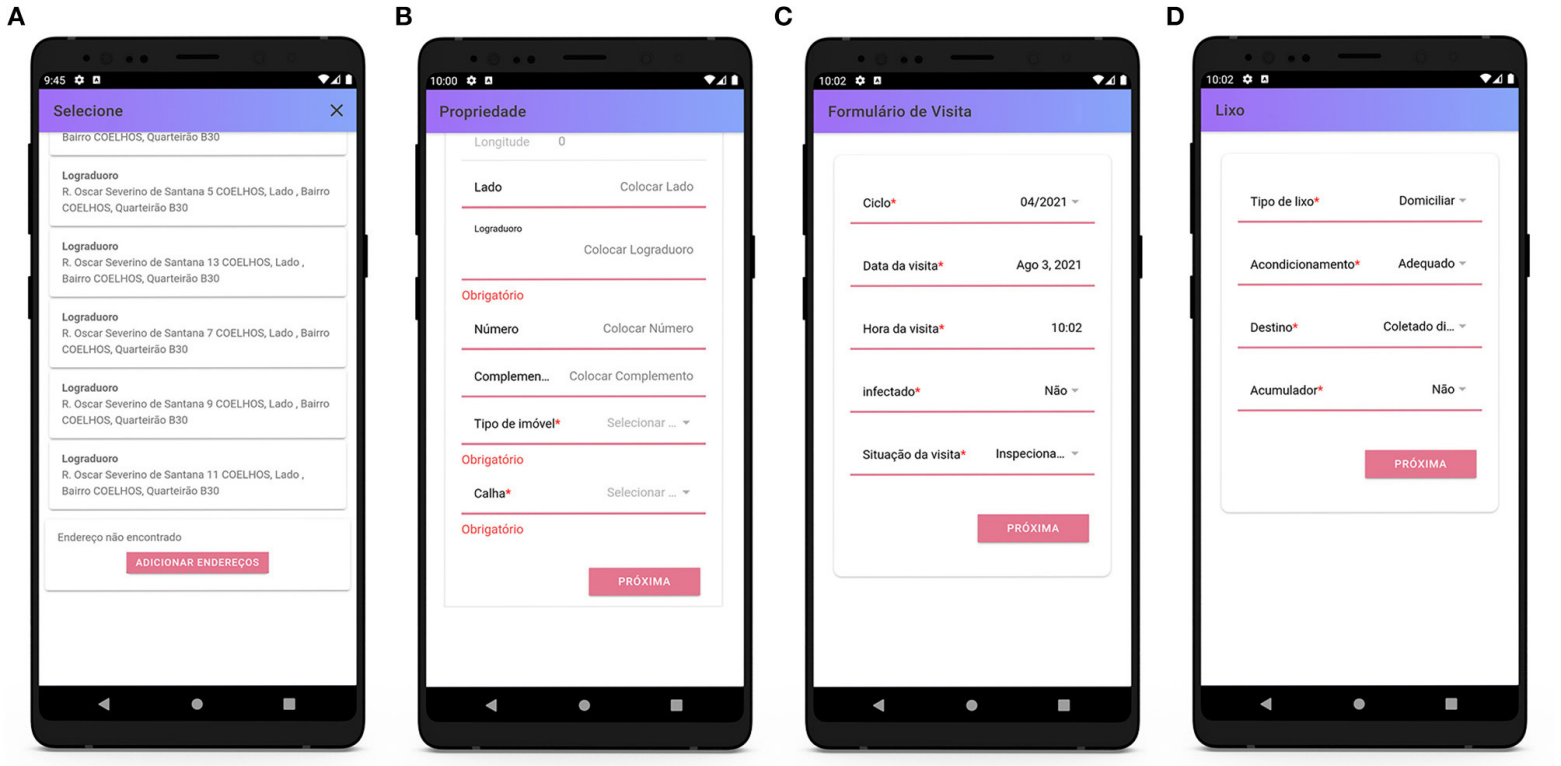
Surveillance agents' mobile application: descriptive and logistical sections of the form. **(A)** List of properties, **(B)** add new property, **(C)** visit form, and **(D)** waste form.

**Figure 8:**
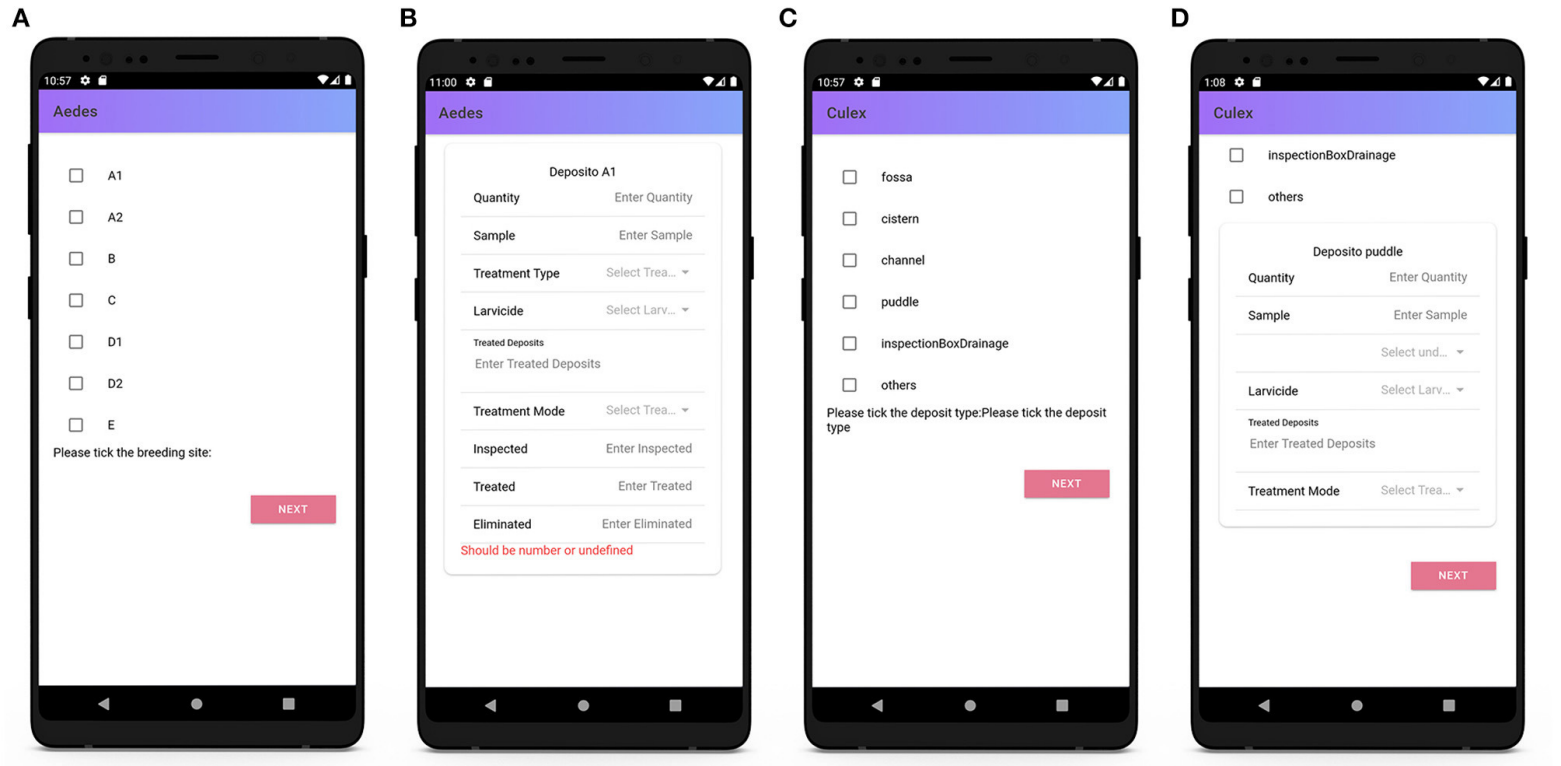
Surveillance agents' mobile application: entomological section of the form. **(A)** Aedes form, **(B)** infected container A1, **(C)** Culex form, and **(D)** infected puddle.

On opening a digital form, the agent selects a property from the list then adds or edits relevant details such as property type (residential, industrial, etc.), the presence (or otherwise) of gutters, and whether the property is infected (Figures 7A,B). If an address is not on the list, the user has the option to add the details of the new address and geographical coordinates using the in-built GPS feature. When the visit is completed, the agent records details such as the time, date, and visit status (completed or not) (Figure 7C), followed by information regarding any of the 13 known types of water container or breeding habitat that are typically located inside or outside dwellings and whether they are contaminated with the larvae of the Aedes or Culex mosquito (the two types of mosquito responsible for the transmission of zika, chikungunya, and dengue), and whether the infected containers have been treated with the appropriate larvicide (Figure 8). Finally, the nature of any environmental waste and waste disposal practices are documented (Figure 7D). Data are transmitted in real time to the cloud-based database. When all properties assigned to an agent have been visited and the forms have been submitted, a task is complete. The manager can then approve (confirm) this completion.

Best practice for the design of mobile forms has been followed to make the process of filling out the forms as straightforward as possible. The more complex a form, the more likely an agent will be to find the process demanding (possibly too demanding) of time and effort. The form is composed of four shorter forms, filled out in a logical order. The amount of typing required has been designed to minimize data entry and so reduce typographical errors. Pre-defined dropdown menus and radio buttons are integrated wherever possible instead of free text. All fields have a validator to help and guide the user if they make an error (such as inputting text when the expected value is a number). All mandatory fields are marked. Finally, because surveillance activities are usually conducted during the summer months when sunlight is at its strongest, a light background with black text has been chosen to improve readability and visibility.

## MEWAR Surveillance System Implementation

The system architecture consists of two main components: the application server and the system client. The server is responsible for providing services to users, while the client side is the interface between the system services and the users. The system has been implemented and developed by applying standard software engineering practices to ensure fewer defects and bugs and to create a high-quality product. These practices include making use of software design patterns to manage the project, using version control to manage multiple system versions and to track changes over time, and using peer code reviews to enhance the code quality (20). The developed system leverages existing technologies such as GitHub, CircleCI, and Heroku for automatic building, testing, and archiving the system. The continuous integration service is essential for improving reliability by minimizing human errors.

### Application Server

The application server is the back end and backbone of the system. The server responds to Hyper Text Transfer Protocol Secure (HTTPS) requests and processes the received data and user authentications. The Node.js with an ExpressJS framework has been used as a server for our system to define the Application Programming Interface (API) endpoints for communication with the database. The advantage of Node.js over other options is the support of asynchronous call-backs, which in turn enhance the response times and smooth the user experience (21). The server is hosted on Heroku with a cloud-based NoSQL unstructured database for data storage. The selection of a database system is crucial to any data-driven software solution. Because the surveillance system and paper forms used in Brazil can differ slightly from city to city and across time, it was reasonable to choose the NoSQL unstructured database (e.g., MongoDB) to avoid restrictions on any future changes to the database models (22). In other words, a structured database would not in practice provide greater flexibility and would enforce the data type in the models (23). It should be noted that the current MEWAR surveillance system service is dedicated only to Recife and Campina Grande surveillance agencies, and data is collected for research purposes only. However, the system is envisioned to support the system's adoption by the two cities for their daily surveillance, scale up to other cities and states, and make it interoperable with state environmental agencies' servers, which require manual report data entries (24).

#### Database Configuration

Having a robust geographical backbone is crucial for the accuracy of any vector surveillance system usually carried out at the household level (10). The database has therefore been initialized with the geographical information concerning both cities, including areas, neighborhoods, and properties. The data regarding areas and neighborhoods was provided by the local Brazilian communities in map format. To ensure compatibility with our system, we turned this data into a JavaScript Object Notation (JSON) format using Geographic Information System (GIS) tools. For the properties, we compiled a list of home addresses from the Brazilian Institute of Geography and Statistics (https://www.ibge.gov.br) and used the Google Geolocation API to find the coordinates of those properties. The Mongoose library has been integrated to manage the relationships between data and to create MongoDB validation as it offers type casting and query building as a built-in solution (25).

### System Client

The system client is the front end of the system where users interact with the system. The client side communicates with the server side through the API calls. The system provides two forms of access: the web interface and the mobile interface. The former is for the managers and supervisors responsible for allocating agents, coordinating their tasks, and monitoring their progress. The mobile application provides health agents with digital forms for recording data during routine surveillance inspection visits matching the precious paper-based forms. Both platforms support the two languages, Portuguese and English.

- **The manager dashboard** has been built with React Native for the web, which is one of the JavaScript libraries that splits UI elements into components which have their own local state. Material-UI, a React UI framework, has been used to provide rich User Interface (UI) functionalities with inbuilt styling. The manager dashboard web page can be accessed through a web browser on any device (desktop, laptop, tablets, mobile, etc.) regardless of platform (Android, iOS, Windows, etc.).
- **The mobile application for agents** has been built in Ionic, a mobile toolkit that compiles code written in either plain JavaScript or any JavaScript framework, such as React (which is used in this system), Angular, or Vue, into Android and iOS apps. The utility of Ionic in relation to the project is the ability to write code once and compile it for both major platforms, iOS and Android. The mobile application also captures spatial data during the fieldwork using the device's Global Positioning System (GPS). The minimum requirement for using the mobile application is a smartphone or tablet supported by either Android or iOS operating systems with access to Wi-Fi or mobile network connectivity.

### Authentication and Security

The API endpoints of our system need to be protected. This implies that there needs to be a way to authenticate a user and ensure that they are only accessing objects linked to their account. For example, an agent should not have access to view other agents' submitted forms, as well as not submit forms for properties that are not assigned to them. Toward this end, a token-based authentication system with two tokens has been implemented: a short-lived authentication token to authenticate the user at every request and a long-lived refresh token to keep users logged in. The tokens are JSON Web Token (JWT) which is defined as an open standard (RFC 7519) used for securely transmitting information between nodes using JSON object (26).

## Discussion and Future Work

The system we have developed is designed to support environmental surveillance agencies in two Brazilian cities, but the underlying conceptual solution and technical infrastructure can be expanded to mosquito surveillance in a wide range of regions. The system enables data to be captured at the finest spatial scale (the household level) on a mobile phone, which in turn yields several other potential advantages such as a reduction in data transcription and transferal errors, real-time quality review, analysis for decision-making, and using the dataset to develop or calibrate models for predicting risk areas.

In addition to the advantages and undoubted operational value the MEWAR system provides to environmental surveillance agents, this study provides valuable insights and knowledge that are essential to the development of similar systems in other settings. What makes this study unique is that it provides a framework for co-design, development, and evaluation based on feedback provided by end users with knowledge gained from a real-world case. Furthermore, the research has brought together experts in computer science, epidemiology, medical entomology, and public health to ensure that the final product boosts the production of target users and minimizes the effects of their limitations. This variety of expertise has helped to address several project challenges and to understand the diverse range of client requirements. The development method presented here is therefore considered generic and transferable to the building of any vector surveillance tool aimed at combating disease by controlling vector in either high or low endemic setting. Moreover, the underlying conceptual solution and technical infrastructure of the system can be easily adapted and localized for other study areas and countries.

The key element of this project research success was the continued equitable involvement of health surveillance agents throughout the project phase and iteratively co-authoring in each research and development phase. However, marrying the research needs and surveillance agents requirements was a challenge on occasion as collecting additional data and variables for the early-warning system modeling had to be scaled down due to the busy schedule of agents who did not have the flexibility to spend extra time collecting data on each property during their allocative scheduled visits. Our prioritized functional requirements were to reduce any discrepancies in data collection by systematically entering the data into the system and having real-time data to support any further analysis. For the agents, rapid entry, and minimizing data entry errors were their priorities. For managers, the ability of the system to facilitate inspection task management and reporting, as well as enhanced decision support, were their prioritized requirements. By reviewing the functional requirements, this work was carried out in a way including of diverse viewpoints yet facilitated consensus.

Because the Covid-19 pandemic temporarily stalled in-field testing activities, the 6-week pilot for data collection is scheduled for mid-August 2021. Once the pilot is running, a qualitative assessment study will be followed in order to evaluate how well the agents adopt the mobile application. However, the result of the first phase of in-house testing demonstrated the engagement of health agents and their willingness to adopt the system for field testing on a large scale where longitudinal surveillance data are collected for training the early warning system, which is the ultimate aim of the MEWAR project. We collaborated from the outset with Brazilian researchers and local surveillance agents and involved them in all phases of product development in order to ensure that health agents will have access to required tech training on using the system. Brazilians currently control the operational use of the system, including the training of health agents, while we at UCL provide engineering and technical support.

Looking forward, in the future version of the system, we aim to improve consistency checks, such as alerts for duplicated visit tasks or assigning more than one agent to the same task. Moreover, we will focus on expanding the main functionalities currently supported on the manager platform, such as allowing the manager to comment on the forms submitted by the agents before issuing an approval or response. Other functionalities include the incorporation of a map linked to a predictive model, enabling the identification of high-risk areas for decision making, and the dynamic allocation of tasks to agents based on risk maps using a built-in allocation algorithm. Any changes to the process of task assignment and data collection might require some redevelopment of the interface. The proliferation of mobile devices makes their use as surveillance tool much easier, however, the network and internet access remain an obstacle indeed, especially in low-income countries (developing countries). Therefore, one way to mitigate this issue is the integration of some caching functionality into the mobile application, which will enable it to operate in areas where there is no internet connection. The caching functionality will allow the mobile application to work in offline mode or when the connection is lost during the process of filling out the forms. Finally, the overarching objective of developing this system is to combine the dataset collected by the mobile application with climate data and larval index data to develop predictive models which can be calibrated to predict the areas most vulnerable to mosquito infestation. The results could be integrated into the manager dashboard as risk map for policymaking, which will result in better decision making regarding the priority of in-field surveillance.

## Conclusion

Mobile technology has enabled the development of significant intervention tools that can enhance the effectiveness of mosquito control campaigns performed by environmental surveillance agencies by switching from the traditional paper-based system to digital forms. Here, we have described the process of designing, developing, and implementing a mosquito surveillance system (MEWAR). The proposed system has the potential to strengthen the entomological surveillance of the mosquito, which is responsible for transmitting several deadly arboviruses in Brazil. The system provides several critical functions that allow workflows to be tracked through a manager platform and provide real-time geolocated reports using a mobile application. Moreover, such a system can be a useful tool for integrating and calibrating models into mosquito surveillance at a fine scale. The proposed system has been developed by following appropriate software design and development practices to improve the quality of the system and reduce maintenance issues. Although the system was designed for environmental surveillance agents in Brazil and is based on that local setting, it could be scaled up to any country, and the knowledge acquired from the developmental process could be transferable to other intervention tools. The system could also be integrated into existing government public health programs that use digital interventions.

## Data Availability Statement

The original contributions presented in the study are included in the article/supplementary material, further inquiries can be directed to the corresponding author/s.

## Author Contributions

TM, WS, and PK were responsible conceptualization of this system. AA was responsible for the original draft. AA, GB, and AB were responsible for developing and implementing the proposed system. AM and PK for substantial revisions. AA, AM, GB, LD, GM, and TM for running and leading the focus groups. GS, VN, and RO were providing invaluable information regarding the fieldwork and testing the system. All authors contributed by supporting the project and providing feedback.

## Funding

This research was conducted under the project a gamified m-training app for health professionals on protocols and participatory surveillance associated with Zika virus funded by British Council Newton Fund No. 280860230 and Mosquito populations modelling for early warning system & rapid public health response (MEWAR) which was funded by the Belmont Forum and supported in the United Kingdom by UKRI NERC under the grant NE/T013664/1. The PhD studentship of the lead author was funded by the Space and Aeronautics Research Institution, National Center for Satellite Technology, King Abdulaziz City for Science and Technology (KACST), Riyadh, Saudi Arabia.

## Conflict of Interest

The authors declare that the research was conducted in the absence of any commercial or financial relationships that could be construed as a potential conflict of interest.

## Publisher's Note

All claims expressed in this article are solely those of the authors and do not necessarily represent those of their affiliated organizations, or those of the publisher, the editors and the reviewers. Any product that may be evaluated in this article, or claim that may be made by its manufacturer, is not guaranteed or endorsed by the publisher.
